# Influence of a Soft Robotic Suit on Metabolic Cost in Long-Distance Level and Inclined Walking

**DOI:** 10.1155/2018/9573951

**Published:** 2018-07-05

**Authors:** Shanhai Jin, Shijie Guo, Hashimoto Kazunobu, Xiaogang Xiong, Motoji Yamamoto

**Affiliations:** ^1^School of Engineering, Yanbian University, Yanji 133002, China; ^2^School of Mechanical Engineering, Hebei University of Technology, Tianjin 300130, China; ^3^Ningbo Institute of Intelligent Manufacturing Industry, Ningbo 315400, China; ^4^School of Mechanical Engineering and Automation, Harbin Institute of Technology Shenzhen Graduate School, Shenzhen 518055, China; ^5^Faculty of Engineering, Kyushu University, Fukuoka 819-0395, Japan

## Abstract

Metabolic cost during walking is positively linked to exercise intensity. For a walking assistive device, one of the major aims should be the maximization of wearers' metabolic benefits for different walking situations. Toward this goal, this paper experimentally evaluates the influence of an authors' soft robotic suit, which has been developed to assist hip flexion for energy-efficient walking of elderly persons in daily life activities, on metabolic cost reduction in the long-distance level and inclined walking. Experiment results show that, for a 79-year-old healthy male subject, the robotic suit significantly reduced metabolic cost in the condition of the robotic suit worn and powered on compared with the condition of worn but powered off.

## 1. Introduction

The proportion of the elderly population is steadily increasing, and the number is estimated to reach almost 22% of the global population in 2050 [1–3]. Many elderly persons suffer walking difficulties because of lower limb skeletal muscle decline caused by aging. As a result, they perform shorter and fewer walking activity, which is positively related to the quality of elderly life, compared with young ones [4]. Such reduced walking activity may result in many psychosocial problems, for example, social isolation, unhappiness, or depression. On the other hand, reduced walking activity, in turn, causes further lower limb skeletal muscle decline. Finally, they may experience a vicious cycle of physical activity reduction and skeletal muscle decline.

To prevent the abovementioned vicious cycle, lower limb exoskeletons have been studied. For example, an exoskeleton for rehabilitating walking function has been produced by Cyberdyne Inc. [5, 6]. As another example, a semiexoskeleton for improving walking ability has been presented by Honda Motor Co. Ltd. [7–9]. Besides these, various lower limb exoskeletons are designed, for example, [10–16]. One major advantage of lower limb exoskeletons is that they are capable of providing sufficiently large assistive force, which may reach several times larger than that produced by human joints [17, 18]. In addition, they can support the full amount or a significant portion of body weight of wearers through rigid frames [19, 20].

However, some challenging problems must be overcome in designing and implementing exoskeletons. (1) Uncomfortable resistive force may be generated between exoskeleton and wearer in the case of axial joint misalignment [21], and thus leading to increased metabolic cost. (2) The motion range of the lower limbs is constrained by rigid frames of the exoskeletons, while conducting daily life activities requires a large motion range [18]. (3) A massive power supply system is required for producing a large assistive force. In addition, the mass attaches an additional payload to the wearer. (4) The procedures of mounting and removing an exoskeleton are complicated. Such problems cannot be ignored in the use of an exoskeleton in daily life activities.

To avoid the drawbacks of exoskeletons, some researchers have researched soft walking assistive devices [22–32]. One of the major advantages of soft robotic suits is that exact matching of device's joints to those of wearer is not required. Besides that, owing to the use of soft materials, they are almost free from kinematic constraints. Additionally, the strength of the provided assistive force is usually less than that of exoskeletons, and thus, the power supply system can be built with smaller size and lighter weight. Moreover, they can realize compatible and safe interactions with wearers [33].

**Figure 1:**
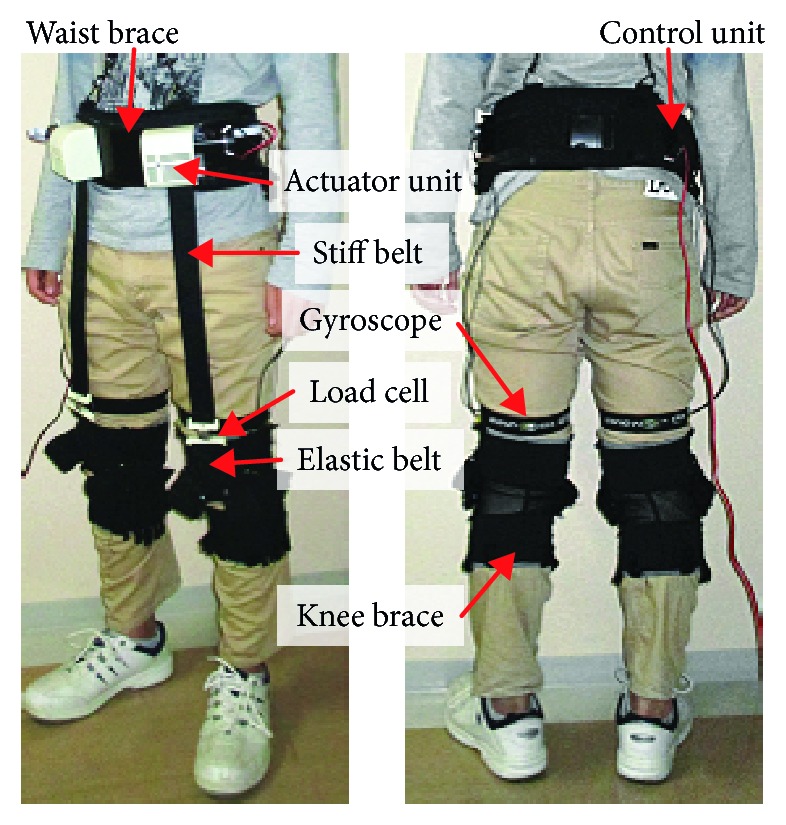
Overall structure of a soft wearable robotic suit.

Specifically, the authors' group has developed a soft robotic suit for energy-efficient walking for elderly persons in daily life activities [29–32], as shown in Figure 1. The robotic suit provides a small but effective assistive force for hip flexion via winding belts that contain elastic elements. Moreover, it is lightweight and it almost does not restrict the motion range of the lower limbs. It is reported [29] that, in the case of 6-minute level walking, the use of the robotic suit in the worn and powered on (PON) condition significantly reduced metabolic cost and significantly improved gait characteristics compared with the condition of worn but powered off (POFF).

One of our final goals is to develop a soft robotic suit that reduces metabolic cost as much as possible for elderly persons during walking in daily life, so that they can conduct more physical activities, for example, shopping, taking a walk, and visiting friends living far, for preventing health care. Toward this goal, this paper experimentally evaluates the influence of the soft robotic suit on metabolic cost of walking in an environment that demonstrates the potential use of the robotic suit in real-life situation.

The rest of this paper is organized as follows.  Section 2 gives an overview of the authors' soft robotic suit.  Section 3 experimentally evaluates the influence of the soft robotic suit on metabolic cost in long-distance level and inclined walking.  Section 4 discusses the experimental results.  Section 5 covers conclusions and future work.

## 2. Overview of a Soft Wearable Robotic Suit

The authors' group has presented a soft wearable robotic suit for energy-efficient walking for elderly persons in daily life activities. Figure 1 illustrates the overall structure of the robotic suit. For each leg, it is composed of one actuator, one control unit, one knee brace, one stiff upper, and one elastic lower belt that are attached to the actuator unit and the knee brace, respectively, one load cell that connects the two belts, and one gyroscope. Here, it should be mentioned that the actuator and the control units, which contain most of the system mass, are mounted to the front and the back of a waist brace, respectively. This is due to the fact that mounting a 4 kg mass to the waist does not significantly increase the metabolic cost, while adding a 4 kg mass to the thigh, shank, or foot expends more metabolic cost compared with the case of waist [34]. The total weight of the robotic suit, excluding the power supply system, is 2.7 kg (the power of the device is externally provided by a DC power source through a cable).

In the robotic suit, undesirable forces generated by disturbances or significant control errors can be absorbed by the elastic elements. Moreover, the robotic suit almost does not restrict the motion range of the lower limbs, and thus wearers can perform a risk-avoiding action in the cases of emergencies. Furthermore, owing to its simple structures, wearers can easily take the robotic suit on and off by themselves.

**Figure 2:**
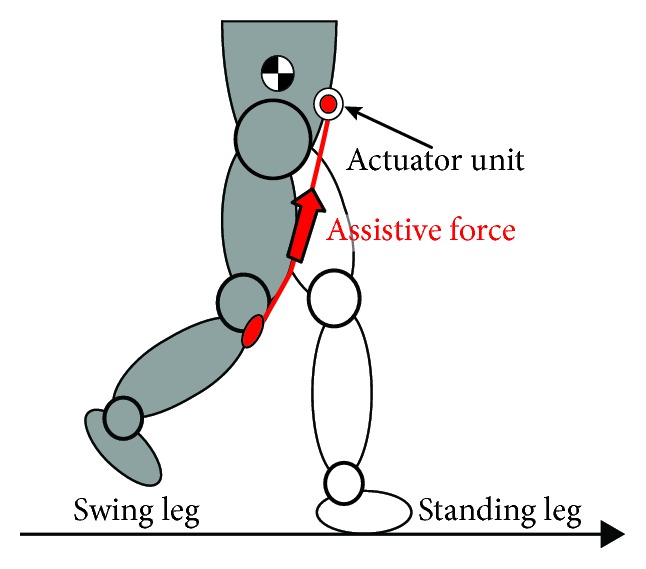
Assistance motion for the swing phase.

The soft robotic suit provides a small but effective assistive force for hip flexion in the sagittal plane of walking. Specifically, as illustrated in Figure 2, during the swing phase, the actuator unit winds up the stiff and elastic belts, and the correspondingly produced tension force on the belt is transmitted to the wearer's joints for assisting hip flexion. On the other hand, during the standing phase, the tension force is maintained in a small but sufficient value (0.6 N) that allows the belts “creeping” along the thigh without influencing the extension of the hip.

**Figure 3:**
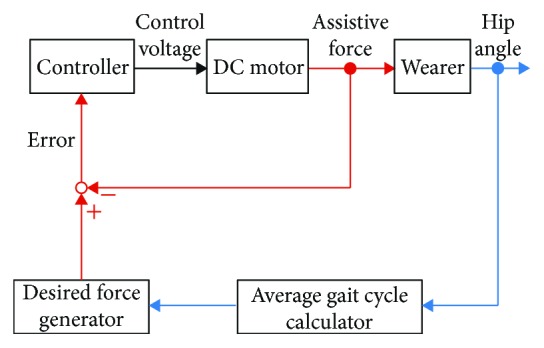
Block diagram of a tension force control scheme.

**Figure 4:**
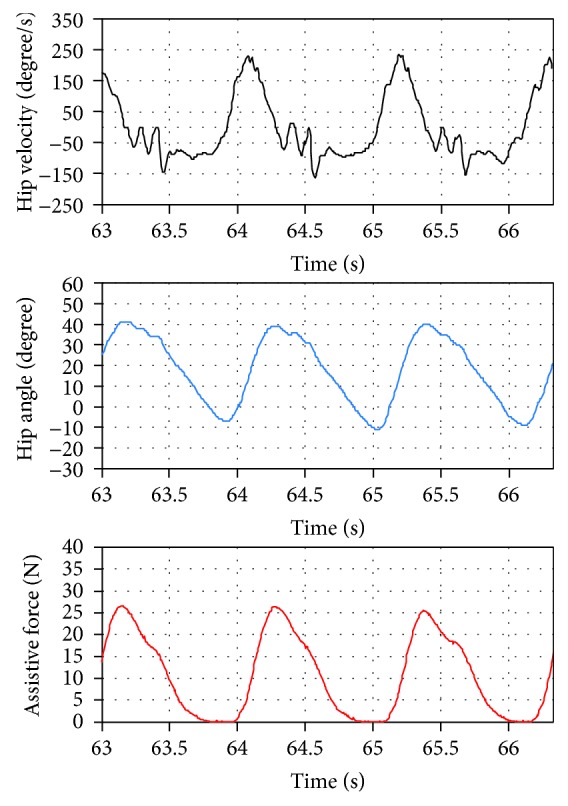
Typical data of hip angular velocity, hip angle, and assistive force.

Figure 3 shows the block diagram of the control system of the soft robotic suit. Hip angular velocity during walking is measured by the gyroscope. Then, it is converted to hip angle by applying numerical integration. It is known that numerical integration of gyroscope velocity signal introduces in angular drift. Toward this problem, a 0-degree angular offset compensation is performed. The timings of minimum hip angle, maximum hip angle and heel contact, and the average gait period are estimated in an average gait cycle calculator, and they are used for generating the desired assistive force profile. A proxy-based sliding mode controller [35], which realizes smooth and safe response [36, 37], is implemented for tracking the desired one with the sampling interval *T* = 0.001 s.  Figure 4 shows typical data of measured hip angular velocity, hip angle, and generated assistive force.

## 3. Experiment

This section experimentally evaluates the influence of the robotic suit on metabolic cost in long-distance level and inclined walking. The experiment was approved by the Experiment Ethics Committee of the Faculty of Engineering, Kyushu University.

### 3.1. Subject

One healthy male elderly subject (age = 79 years, weight = 61.6 kg, and height = 157 cm) participated in the experiment.

### 3.2. Protocol

Before the main experiment, a preliminary exercise was performed for the subject. First, the subject was instructed to familiarize himself with treadmill walking. Then, his preferred walking speed (2.2 km/h) was evaluated by applying the procedure reported in [38]. After that, the subject practiced the treadmill walking in the PON condition with the maximum assistive force (MAF) 25.3 N that he felt comfortable at the preferred walking speed for getting used to the robotic suit.

**Figure 5:**
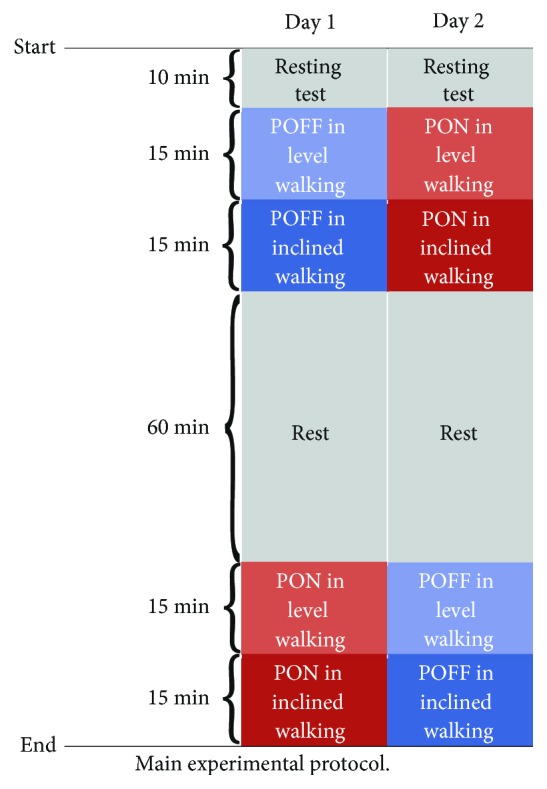
Main experimental protocol.

The experiment was performed in two days.  Figure 5 shows the main experimental protocol. A 30-minute treadmill walking trial at the preferred speed with the POFF condition versus the PON condition constituted one set of the comparative experiment. On each experimental day, one set of comparison was performed. For each trial, firstly the subject conducted a level walking for 15 minutes. After that, the treadmill was inclined to 2% slope by the experimenter, and the subject performed an inclined walking for the left 15 minutes. Specifically, in the case of the PON condition, the same assistive force profile, as illustrated in Figure 4, was applied for both level and inclined walking. A 10-minute resting test was performed for determining the resting metabolic cost. In addition, a 60-minute rest period was provided after trial 1 for both recovering metabolic activity and separating of the two trials. To exclude the influence of metabolic and biomechanical variations and measurement errors, reversed orders of the POFF condition and PON condition were used on different days. Here, it should be mentioned that, because the purpose of the experimental protocol was to validate the effectiveness of walking assistance strategy, that is, assistance for hip flexion via winding belts, during long-distance level and inclined walking, and since the actuator unit was not optimized for weight, only the comparison between the POFF condition and the PON condition was considered, excluding the condition of the robotic suit not worn.

Expired gas was collected continuously by a gas analyzer (AT-1100, Anima, Co., Japan) for the entire 30-minute walking.

### 3.3. Data Analysis

For each day, average values of resting metabolic cost ${\dot{V}}_{O2\left(rest\right)}$ (ml/min) during the last 5-minute interval of resting test were calculated for determining the resting metabolic level. In addition, for all trials, average values of gross walking metabolic cost ${\dot{V}}_{O2\left(gross\right)}$ (ml/min) in both 15-minute level walking and 15-minute inclined walking were computed. Moreover, average values of ${\dot{V}}_{O2\left(gross\right)}$ over the entire 30-minute interval were calculated. Besides that, for each trial, in order to examine the transition of metabolic cost, average values of ${\dot{V}}_{O2\left(gross\right)}$ per 5-minute interval were computed. Then, average values of net metabolic cost ${\dot{V}}_{O2\left(net\right)}$ (ml/min) were obtained by subtracting corresponding ${\dot{V}}_{O2\left(rest\right)}$ from the abovementioned each ${\dot{V}}_{O2\left(gross\right)}$. After that, ${\dot{V}}_{O2\left(net\right)}$ was normalized by body weight (kg). The averages of all the trials in each measure were used for the analysis.

### 3.4. Statistical Analysis

Paired *t*-tests were performed to identify the significant differences between the POFF condition and the PON condition in averaged ${\dot{V}}_{O2\left(net\right)}$ of 15-minute level walking, 15-minute inclined walking, and entire 30-minute walking. In addition, standard deviations were computed for each averaged ${\dot{V}}_{O2\left(net\right)}$.

## 4. Results

**Figure 6:**
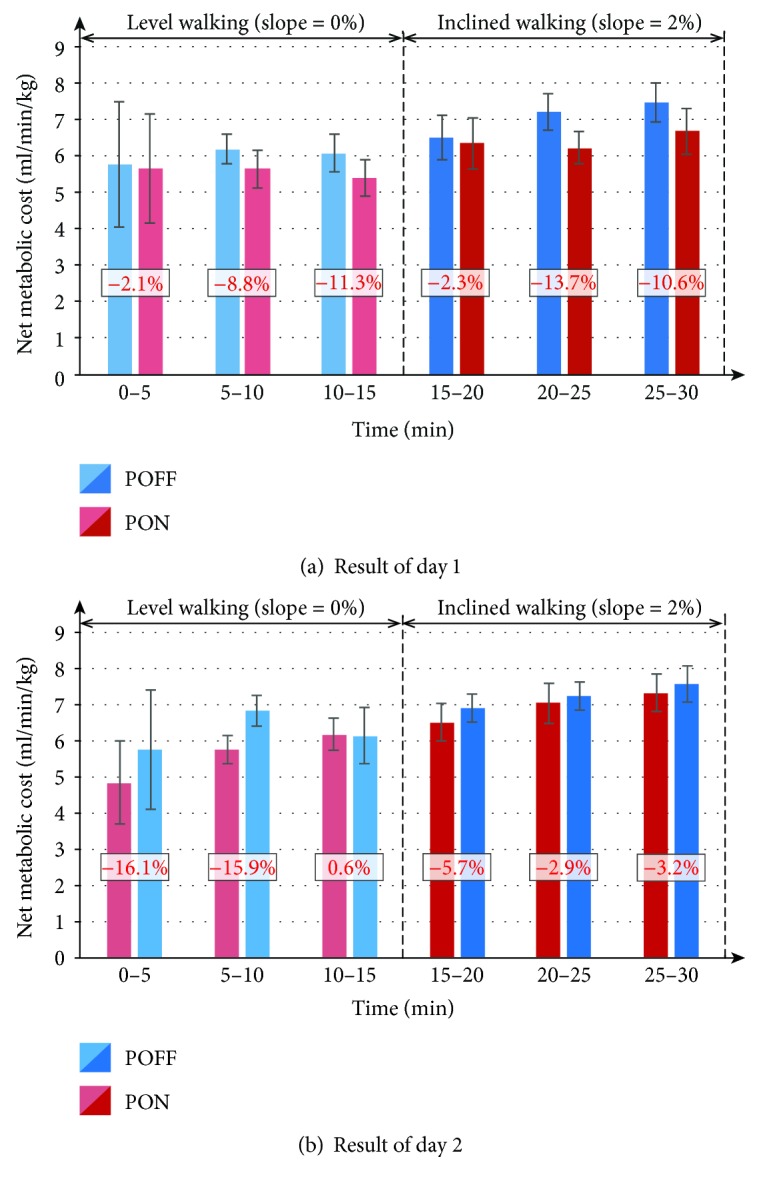
Comparison of metabolic cost between the POFF condition and the PON condition for 5-minute intervals. On each bar chart, the error bar denotes standard deviation. In addition, the number expressed in percentage represents the difference of the PON condition compared with the POFF condition.

Figure 6 shows the metabolic costs achieved in the POFF condition and the PON condition for each 5-minute intervals. Metabolic cost was reduced in the PON condition in eleven of twelve intervals compared with the POFF condition, with a maximum reduction of 16.1%.

**Figure 7:**
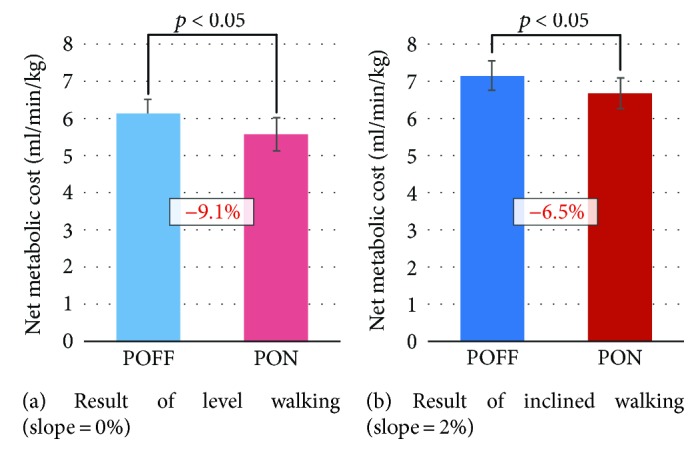
Comparisons of two-day average value of metabolic cost between the POFF condition and the PON condition for 15-minute level walking and 15-minute inclined walking. On each bar chart, the error bar denotes standard deviation. In addition, the number expressed in percentage represents the difference of the PON condition compared with the POFF condition.

Figure 7 compares the two-day average value of metabolic cost between the two conditions in terms of level walking and inclined walking. One can observe that, for both cases, statistically significant differences were found between the two conditions, with averaged 9.1% and 6.5% reductions in the PON condition.

**Figure 8:**
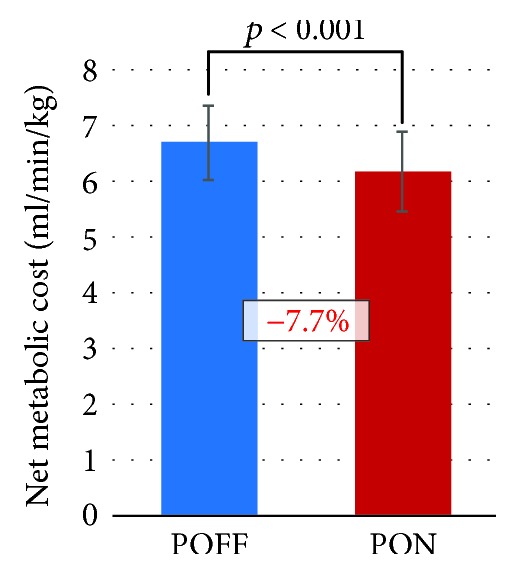
Comparison of two-day average value of metabolic cost between the POFF condition and the PON condition for entire 30-minute walking. On each bar chart, the error bar denotes standard deviation. In addition, the number expressed in percentage represents the difference of the PON condition compared with the POFF condition.

Two-day average value of metabolic cost of entire 30-minute walking was compared in Figure 8. It is shown that the use of the robotic suit significantly reduced metabolic cost by an average of 7.7%.

## 5. Discussion

One of the final aims of the robotic suit is to reduce metabolic cost as much as possible for elderly persons in daily life activities. As illustrated by the results, metabolic cost of level walking was reduced in the PON condition compared with the POFF condition for almost every 5-minute intervals, with a maximum reduction of 16.1% and an average reduction of 9.1%. This result is consistent with our previous result [29] showing that the use of the robotic suit reduced metabolic cost with an average of 5.9% during 6-minute level walking. However, it is known that metabolic stress of walking increases as walking duration increases [39–42]. Thus, it should be highlighted that, compared with the previous result of 6-minute walking that might be too short for imposing significant metabolic stress on the subject, the reduction of metabolic cost during the 15-minute walking was achieved under the condition of longer continuous accumulation of metabolic stress. The major underlying cause of this reduction was probably owing to the continuous energy injection of the robotic suit for assisting the hip flexion, that is, metabolic cost reduction obtained by mechanical energy injection.

In the case of inclined walking, metabolic cost was higher than the case of level walking for both conditions. This is primarily due to the fact that, in the case of inclined walking, the human body has to be raised against gravity by increasing hip flexion as gradient increases [43–45]. Thus, additional mechanical power might be consumed in the hip joint for increasing the gravitational potential energy of the body's center of mass (COM) compared with the level walking [43, 46, 47]. Besides that, the metabolic stress accumulated through the previous 15-minute level walking was also probably linked to the increased metabolic cost of the inclined walking. However, by comparing the results of the two conditions, one can found that metabolic cost of the PON condition was lower than that of the POFF condition, with a maximum reduction of 13.7% and an average reduction of 6.5%. Toward such a reduction, we assume that the provided hip flexion assistance contributed to the hip flexion strength by compensating the metabolic burden of the hip joint during the inclined walking and consequently led to the reduced metabolic cost of the PON condition.

Interestingly, it should be observed that the metabolic reduction rate of inclined walking was less than that of level walking. From this result, we suppose that, under the same injected power, that is, the same assistive force profile parameters, the injected power during inclined walking was not sufficient for compensating the increased mechanical power required to accelerate COM upward, achieving a similar metabolic reduction as the case of level walking. This finding suggests that, in order to maximize the effectiveness of the robotic suit, optimized assistive force profile parameters, for example, shape, MAF, and timings of start, MAF, and end, should be explored by getting a better understanding of complex human-machine interaction.

As a whole, since metabolic cost is positively related to exercise intensity [48], we argue that the subject probably walked more easily and comfortably in the PON condition with the less metabolic cost during the 30-minute level and inclined walking. Thus, it can be concluded that the robotic suit is effective not only in short-distance level walking as reported in the previous work [29], but also in long-distance level and inclined walking.

## 6. Conclusions and Future Work

This paper has experimentally evaluated the influence of the authors' soft robotic suit on metabolic cost in 30-minute level and inclined walking. Experimental results show that metabolic cost reduced with a maximum reduction of 16.1% in the PON condition compared with the POFF condition for eleven of twelve 5-minute intervals. In addition, significant metabolic cost reductions of 9.1% and 6.5% were found in the PON condition for level walking and inclined walking, respectively. Moreover, for the entire 30-minute walking, the robotic suit significantly reduced metabolic cost by an average of 7.7%.

One limitation of this study is that, by considering the 2.7 kg mass of the robotic suit additionally imposed to the wearer, the effectiveness of the robotic suit was only compared between the POFF condition and the PON condition. Future study includes optimizations of mechanical structure (including weight reduction), assistive force profile parameters (including shape, MAF, and timings of start, MAF, and end), and design of adaptive control scheme for specific populations and walking environments with the aim of achieving greater metabolic cost reduction and compliant human-machine interaction.
